# Lytic bacteriophages affect the population dynamics of multi-strain microbial communities

**DOI:** 10.20517/mrr.2023.20

**Published:** 2023-09-05

**Authors:** Maciej Spus, Yohanes Raditya Wardhana, Judith C.M. Wolkers-Rooijackers, Tjakko Abee, Eddy J. Smid

**Affiliations:** ^1^TI Food and Nutrition, Wageningen 6700 AA, the Netherlands.; ^2^Food Microbiology, Wageningen University, Wageningen 6700 AA, the Netherlands.

**Keywords:** Microbial community, bacteriophage, population dynamics, starter culture

## Abstract

**Background:** Lytic bacteriophages infect and lyse bacteria and, as a by-product, may affect diversity in microbial communities through selective predation on abundant bacterial strains. We used a complex dairy starter named Ur to investigate population dynamics of *Lactococcus lactis*, *Lactococcus cremoris* and *Leuconostoc mesenteroides* strains in terms of constant-diversity and periodic selection models.

**Methods:** To mimic the starter Ur, we designed blends of 24 strains representing all eight previously identified genetic lineages in the starter culture. The blends were propagated by daily transfers in milk for over 500 generations in the presence or absence of a cocktail of lytic bacteriophages. The relative abundance of genetic lineages of *L. lactis*, *L. cremoris* and *Lc. mesenteroides* strains present in the complex blend, as well as phage presence, were monitored.

**Results:** Control blends without phage predation showed decreased strain diversity, leading to a stable state due to the domination of the fittest strain(s) of a particular lineage according to periodic selection dynamics. However, in phage-challenged blends, predation caused a large shift in the microbial composition by killing the fittest and sensitive strains.

**Conclusion:** It was demonstrated that phage-challenged blends maintained their diversity at the level of genetic lineages, thus providing experimental support for the constant-diversity dynamics model in a complex microbial community.

## INTRODUCTION

In natural environments, bacteria rarely or never exist as a homogeneous single-strain culture but rather as microbial consortia encompassing many strains representing different lineages of a variety of species^[1]^. The species richness and strain diversity are a function of the physico-chemical and biological conditions found in a given habitat. However, bacteria not only interact with each other and their environment but are also typically exposed to bacteriophage predation^[2]^.

Erkus *et al*. characterized in detail an industrially relevant microbial community of a complex cheese starter culture named *Ur*^[3]^. As highlighted by Smid *et al*., the history of use of the *Ur* starter led to the establishment of, at first glance, a simple three-species (*Lactococcus lactis*, *Lactococcus cremoris* and *Leuconostoc mesenteroides*) culture^[4]^. Further analysis of the *Ur* starter demonstrated a substantial degree of diversity beyond the sub-species level. In fact, seven genetic lineages of *Lactococcus* and an eighth lineage of *Lc. mesenteroides* could be distinguished by amplified fragment length polymorphism (AFLP) typing within a representative collection of single strain isolates. Bacteriophage resistance tests with individual strains^[5]^ uncovered another level of diversity among isolated strains of the *Ur* starter belonging to the same genetic lineage. Interestingly, the sensitivity for lytic phages among strains belonging to the same genetic lineage varied substantially^[5]^.

In general, bacteriophages are thought to play a crucial role in controlling the abundance of bacteria in the environment^[6,7]^. A theoretical model explaining the role of bacteriophages in microbial communities was proposed previously by Thingstad^[8]^, who suggested that the “Kill-the-Winner” (KtW) principle explains microbial diversity in aquatic microbial systems. This model explains the prevention of niche domination by the best competitors and thus the maintenance of community diversity. Along with the principles set by Thingstad^[8]^, Rodriguez-Valera *et al*. introduced the constant-diversity (CD) dynamics model, where phage predation is a driver of microbial communities’ diversity^[9]^. The CD dynamics model is different from the periodic selection (PS) dynamics model, although both models are not mutually exclusive. In the case of PS dynamics, the fittest strain eventually dominates the niche, which leads to a clonal sweep of other less fit strains or lineages. The clonal sweep is expected to result in lower ecosystem efficiency as indicated by Rodriguez-Valera *et al*.^[9]^. If PS dynamics take place in the community of a complex starter culture, it may affect the starter’s functionality. The CD model explains the generation and maintenance of microbial diversity in natural ecosystems where bacterial populations can interact with each other and with lytic bacteriophages. In such habitats, nutrients are dissolved and bacterial populations are characterized by large diversity in bacteriophage resistance. Diversity in bacteriophage resistance prevents lysis of the complete bacterial population caused by bacteriophages due to the presence and emergence of resistant variants. According to Rodriguez-Valera *et al*. not only bacterial populations in aquatic environments could follow CD dynamics but also other communities of interacting microbes found in nature^[9]^.

All natural complex systems are exposed to gradual changes in abiotic and biotic conditions. A smooth response to such changes can be disrupted by a sudden catastrophic shift, resulting in an alternative state. Such shifts have been observed in natural complex systems such as lakes, coral reefs, and oceans^[10-12]^. Various triggers can cause these shifts, eventually leading to the loss of resilience (capacity to respond to a change) which is a factor pushing a microbial community towards a tipping point of the shift. The shift in community composition leads to an alternative state that is difficult to reverse. Often, catastrophic shifts are caused by a stochastic event, which can lead to, e.g., a wipeout of a part of a population^[13]^. In the particular case of a complex starter culture, such a wipeout of a sub-population can be caused by bacteriophage predation. The negative impact of phage predation on acidification during the production of dairy products has previously been covered by various publications^[14,15]^. Nevertheless, complex starter cultures, in general, present better resilience to phage predation as compared to defined starters^[16]^. Several studies describe the impact of bacteriophages on individual bacterial strains^[17-19]^, but in the case of complex (multi-strain) microbial communities, empirical data that could support the PS and/or CD dynamic community models^[9]^ are completely lacking. Rodriguez-Valera *et al*. suggested experimentally measuring the individual fitness of different bacterial strains isolated from a natural habitat to determine whether fitter variants are selected against environments under phage pressure^[9]^.

Therefore, in this study, we used complex blends of well-characterized strains from the *Ur* starter as a model system to investigate the role of bacteriophages in population dynamics. These blends were designed according to the following criteria. Firstly, all eight genetic lineages of the complex dairy starter culture called *Ur*^[3]^ were represented by multiple strains. Secondly, all strains were mixed at equal initial relative abundance. Thirdly, we deliberately included strains with different bacteriophage resistance profiles^[5]^. Consequently, we obtained a blend of strains that resembled the diversity of the natural complex starter culture at the level of (i) genetic lineages; (ii) at the strain level; and (iii) at the different levels of bacteriophage resistance. Our blends were sequentially propagated in milk for more than 500 generations without (control) or with the addition of a phage cocktail composed of three lytic phages isolated previously from the *Ur* culture^[3]^. Throughout the propagation experiment, we monitored the abundance of genetic lineages and the presence of bacteriophages. Our study generated empirical data on phage predation and microbial population dynamics that were subsequently analyzed in the context of PS and CD dynamic community models.

## METHODS

### Preparation of the multi-strain blend

The starter culture *Ur* (obtained from foundation BOZ, Ede, the Netherlands) is comprised of three species of lactic acid bacteria (LAB): *Lactococcus lactis*, *Lactococcus cremoris*, and *Leuconostoc mesenteroides*. Many single colony isolates of the *Ur* culture were previously characterized using AFLP-typing^[20]^ and ascribed to eight genetic lineages^[3]^. Five genetic lineages (1, 3, 5, 6, and 7) belong to the species *L. cremoris*, two lineages (2 and 4) belong to the species *L. lactis* (*L. lactis* ssp. *lactis* biovar diacetylactis), and one (lineage 8) is identified as *Lc. mesenteroides* ssp. *cremoris*. Genetic lineages of the *Ur* starter possess traits that are relevant for their function in converting milk into cheese. For instance, strains of *L. cremoris* lineages 1, 3 and 5 have caseinolytic activity linked to the presence of a functional extracellular protease, which cleaves caseins into peptides (prt^+^)^[21,22]^. Moreover, strains of *L. lactis* lineages 2, 4 and *Lc. mesenteroides* lineage 8 are able to convert citrate (cit^+^) into acetoin and/or diacetyl^[23]^, two compounds that have a profound impact on cheese aroma. The main function of the remaining two *L. cremoris* lineages 6 and 7 is the fast conversion of lactose causing rapid acidification of the cheese milk^[3]^.

When the genomes of representative strains of *L. cremoris* lineages 1 and 5 and *L. lactis* lineages 2 and 4 were compared, only a limited number of unique gene sequences was found between the strains in these two genome pairs. None of these unique genes met the criteria for specific primer design [to be potentially used to differentiate them by (q)PCR], and thus *L. cremoris* lineages 1 and 5 (1 & 5) and *L. lactis* lineages 2 and 4 (2 & 4) were further identified and enumerated together^[3]^.

**Figure 1:**
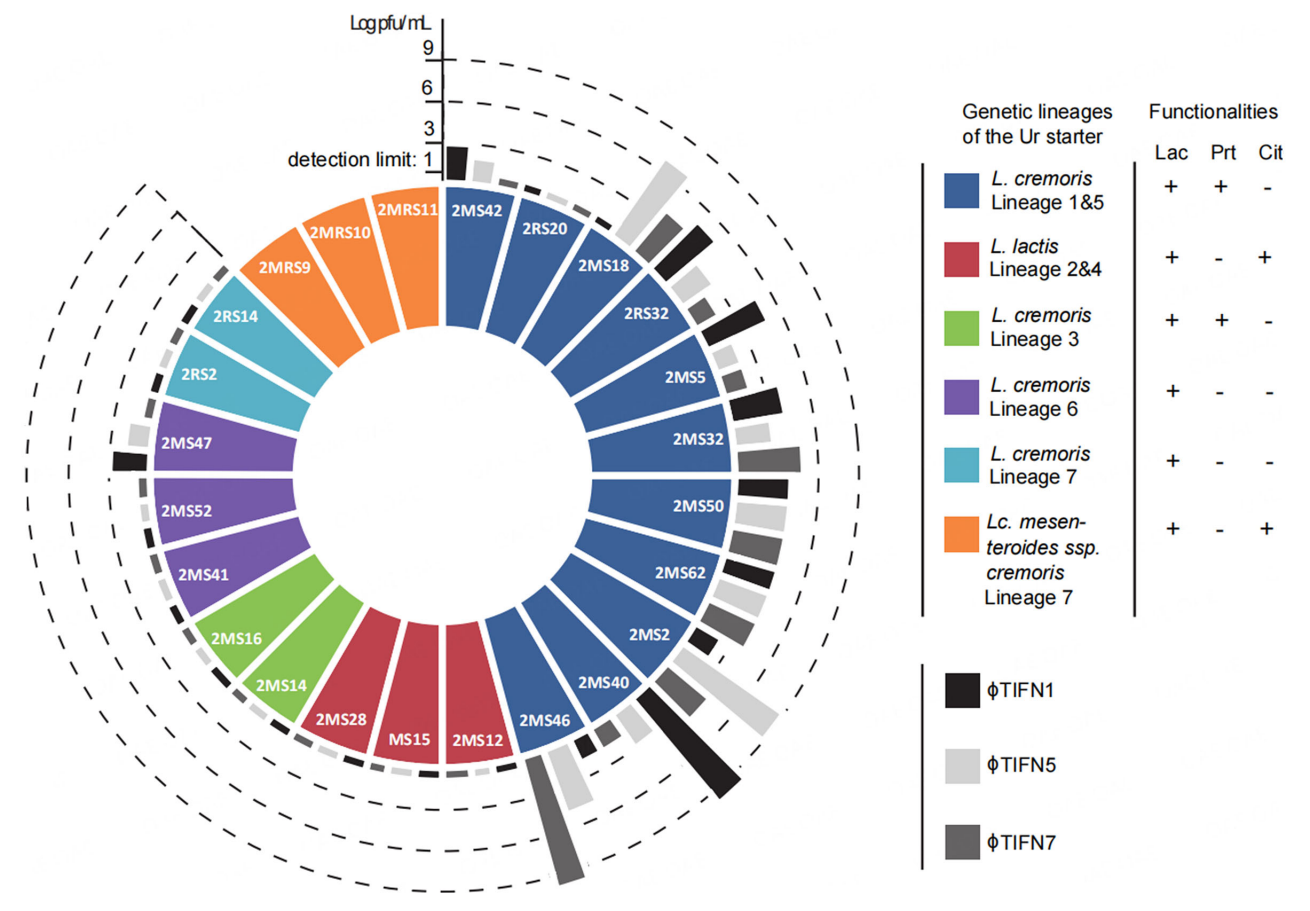
Design of the multi-strain blend used in the propagation experiment. *Lactococcus lactis*, *Lactococcus cremoris* and *Leuconostoc mesenteroides* were represented with 24 strains from eight genetic lineages of the Ur starter culture. The 24 strains are visualized in the first (inner) circle of the diagram where each color indicates a different genetic lineage. For bacteriophage challenge, a cocktail of three bacteriophages was used including lactococcal phage ϕTIFN1, ϕTIFN5 and ϕTIFN7. Strains in the blend differ among each other in terms of resistance to phages present in the cocktail - illustrated by black, dark grey and light grey columns in the second (outer) circle of the diagram. Lineage 8 encompasses *Lc. mesenteroides* ssp. *cremoris* strains, which are resistant to lactococcal phages used in this study. Cit: Citrate degradation ability; Lac: lactose utilization ability; Prt: caseinolytic protease activity.

The blend design included 24 strains representing all eight genetic lineages of the *Ur* starter: eleven strains belonging to *L. cremoris* lineages 1 & 5, three strains belonging to *L. lactis* lineages 2 & 4, two strains of *L. cremoris* lineage 3, three of *L. cremoris* lineage 6, two of *L. cremoris* lineage 7 and finally three belonging to *Lc. mesenteroides* lineage 8 [Figure 1]. To prepare the strains for the blend preparation, frozen cultures of *L. lactis* and *L. cremoris* were streaked on M17 agar (OXOID, Basingstoke, UK) plates supplemented with 0.5% (wt/vol) lactose (LM17) and *Lc. mesenteroides* strains were streaked on MRS agar plates (1.5% agar, Merck KGaA, Darmstadt, Germany) supplemented with vancomycin (final concentration of 30 μg/mL). Next, single colonies were picked and transferred to LM17 broth (in cases of *L. lactis* and *L. cremoris* strains) and MRS broth (in cases of *Lc. mesenteroides* strains). Cultures of *L. lactis* and *L. cremoris* strains were incubated at 30 °C for 18 h (overnight) and cultures of *Lc. mesenteroides* were incubated at 25 °C for 18 h (overnight). Cells were washed twice by removing the supernatant after centrifugation at 2,500 × *g* for 30 min and exchanging it with peptone physiological saline (PPS). The optical density of washed cell preparations was determined at 600 nm (OD_600_) (Novaspec II, Pharmacia Biotech, Pharmacia LKB, Montreal, Canada). The cell suspensions were diluted to OD_600_ = 0.4 (st. dev. ± 0.02) and 100 µL of each suspension was then mixed to obtain the blend to inoculate milk for the propagation experiment. After the blend preparation, the relative abundance of each genetic lineage was determined using a qPCR approach (described below). The initial ratio of the genetic lineages was determined to be as follows: 49.5% of *L. cremoris* lineage 1 & 5, 12.5% of *L. lactis* lineage 2 & 4, 8% of *L. cremoris* lineage 3, 15.5% of *L. cremoris* lineage 6, 12.5% of *L. cremoris* lineage 7 and 2% of *Lc. mesenteroides* lineage 8. Strains used to create blends were previously characterized in terms of their bacteriophage resistance^[5]^. The levels of resistance to three bacteriophages [*Lactococcus* phage ϕTIFN1 and *Lactococcus* phage ϕTIFN7 - both P335 lytic phages and *Lactococcus* phage ϕTIFN5 (a 936 lytic phage)] used in the cocktail to challenge the blends are indicated in Figure 1. Eleven strains included in the blend showed diverse susceptibility to at least one of the three phages and ten out of these eleven were ascribed to *L. cremoris* lineage 1 & 5 and one to *L. cremoris* lineage 6 (strain 2MS47). In total, four blends (two sets of two biological replicates) were prepared: A-1 and A-2; control, no phage cocktail added; B-1 and B-2; treatment, cocktail of three phages added (see Graphical Abstract of the paper). At the onset of the propagation experiment, a cocktail of three phages, ϕTIFN1, ϕTIFN5, and ϕTIFN7, was added to two (treatments) out of four replicates (final concentration: 10^8^ pfu/mL per phage type). As previously reported based on sequence analysis^[3]^, phages ϕTIFN1 and ϕTIFN7 belong to p335-type lactococcal bacteriophages, and phage ϕTIFN5 belongs to 936-type. Both lactococcal bacteriophages are strictly lytic^[24,25]^.

### Propagation regime of the blends

The blends were cultivated in skim milk (“Friesche Vlag Lang Lekker”, non-fat, UHT, FrieslandCampina, the Netherlands) for 24 h. After 24 h, 1% (0.1 mL) of the culture was transferred to fresh milk (9.9 mL). Consequently, each transfer cycle corresponds to approximately 6.64 generations. The experiment was performed continuously for 81 days, which approximately corresponds to 538 generations. Samples of propagated blends were collected after one day (6.64 generations) and next after every 20 days (each 132.8 generations).

### Unique-gene based qPCR for monitoring the relative abundance of genetic lineages

The unique-gene based qPCR method developed by Erkus *et al*.^[3]^ and described in detail in^[5]^ was used to monitor the relative abundance of the genetic lineages. Each sample was measured in duplicate, and for each measurement, a melting curve was monitored to check for unspecific product amplification. The relative abundances of *L. cremoris* lineages 1 & 5, 3, 6, 7, *L. lactis* lineages 2 & 4 and *Lc. mesenteroides* lineage 8 were determined after 6.6, 139, 272, 405 and 538 generations. The Shannon diversity index^[26]^ at the level of genetic lineages was calculated using the following equation: -Ʃ*_i_*[*n_i_*/*N* × ln(*n_i_*/*N*)], with *n_i_* - the abundance of lineage *i* and *N* - the total lineages abundance. In this case, a higher Shannon diversity index indicates a more equal distribution of the genetic lineages.

### Phage-resistance assay

To test if the strains used in blends evolved resistance or to screen for possibly induced prophages during the course of the experiment, we used a qualitative spot assay^[27]^. The supernatants of the blends at different time points (after 6.6, 139, 272, 405, and 538 generations) were used as a possible source of phages and spotted onto the bacterial cell lawn of each of the strains used in the blend preparation. In detail, 10 µL of filtered supernatant (0.2 µm pore size sterile Minisart® filters, Sartorius Stendim Biotech, Göttingen, Germany) was used to spot each indicator culture in the soft agar layer (0.75% agar w/v; 0.5% lactose; 10 mM CaCl_2_).

The same procedure was performed with the end time point (after 538 generations) isolates (200 single colony isolates, 50 isolates per replicate) using the filtered supernatant as well as the undiluted solution (10^10^ pfu/mL) of all three bacteriophages used in the cocktail (ϕTIFN1, ϕTIFN5, and ϕTIFN7). To obtain single colony isolates from the propagated cultures, all four blends were diluted in PPS and plated onto LM17 agar. After 48 h of incubation at 30 °C, 50 single colonies of each blend were picked from the plates, transferred to LM17 broth, and incubated for 24 h. These new single colony isolates were preserved at -80 °C in glycerol (20% v/v) for further use.

## RESULTS

### Population dynamics in the multi-strain blends

**Figure 2:**
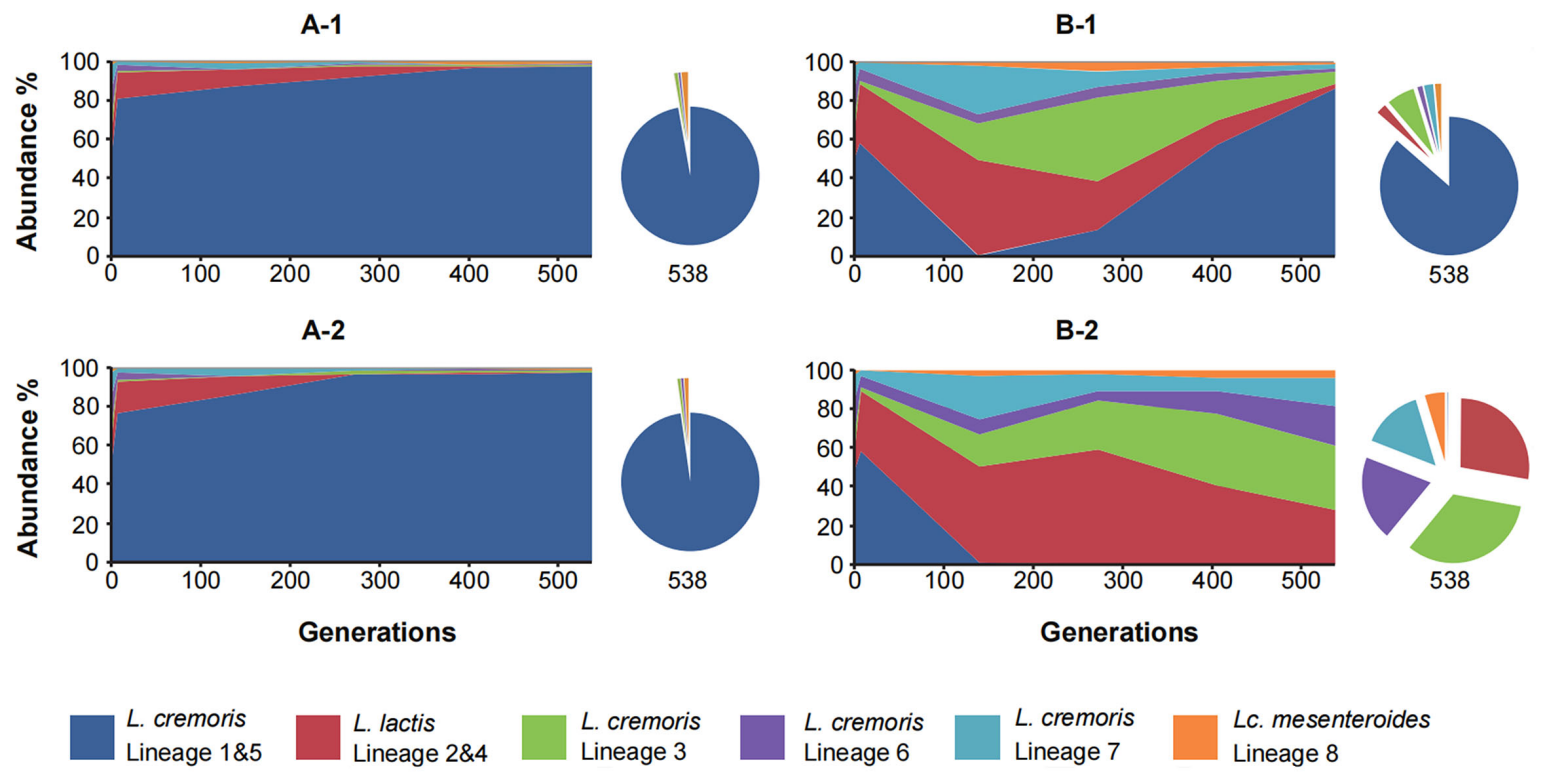
Relative abundance in percentage of lineages in blends during the propagation experiment based on DNA copy number values obtained with unique-gene-based qPCR. Panel A-1 represents the 1st replicate of the blend without phage challenge; Panel A-2 represents the 2nd replicate of the blend without phage challenge; Panel B-1 represents the 1st replicate of the blend with phage challenge; Panel B-2 represents the 2nd replicate of the blend with phage challenge.

Strains of *L. cremoris* genetic lineage 1 & 5 used for the blend preparation were selected for their varying levels of bacteriophage sensitivity/resistance [Figure 1]. For strains of the other lineages used in the blend, with one exception of strain 2MS47 (*L. cremoris*, lineage 6), we did not detect susceptibility to any of the three *Lactococcus* phages (ϕTIFN1, ϕTIFN5 and ϕTIFN7) used in the cocktail. To evaluate the impact of the phage presence/absence, we monitored the abundance of all genetic lineages in both control (A-1, A-2) and phage-challenged blends (B-1, B-2) throughout a long-term propagation experiment [Figure 2]. At the start of the experiment, the distribution of abundance of representatives of the genetic lineages was the same in all four cultures. This initial composition was not stable throughout the propagation experiment; in control blends, we observed a gradual increase in the abundance of *L. cremoris* lineage 1 & 5 strains. At the end time point of the experiment, this gradual increase resulted in the dominance of the *L. cremoris* lineage 1 & 5 strains in both control blends, with a relative abundance of 97% in *L. cremoris* strains A-1 and 98% in A-2. In the phage-challenged blends, we observed a far more complex behavior in terms of dynamics of relative abundances of all genetic lineages. Until 139 generations, both replicates B-1 and B-2 behaved in a similar way - the abundance of *L. cremoris* lineage 1 & 5 strains decreased to a level below 1%, which is in line with the fact that most (10/11) of the strains of *L. cremoris* lineage 1 & 5 are susceptible to at least one of the bacteriophages used in the cocktail. At the next sampling point (272 generations) the abundance of *L. cremoris* lineage 1 & 5 strains in B-2 was still very low, below 0.5% and *L. lactis* lineage 2 & 4 strains (cit^+^) were found to be the most dominant with 59% relative abundance, with *L. cremoris* lineage 3 strains (prt^+^) as the second most abundant (25%) followed by *L. cremoris* lineage 7 strains (prt^-^ and cit^-^) with 9% and *L. cremoris* lineage 6 strains (prt^-^ and cit^-^) with 4.5% abundance. Finally, *Lc. mesenteroides* ssp. *cremoris* lineage 8 strains (prt^-^ and cit^+^) occupied 2.5% of the total population. However, in the other replicate B-1 at 272 generations, *L. cremoris* lineage 1 & 5 strains increased in abundance to 13%, with the other prt^+^ *L. cremoris* lineage 3 strains as the most dominant at this point with 44%, followed by *L. lactis* lineage 2 & 4 strains with 25% and *L. cremoris* lineage 7 strains with 8% relative abundance. Previous experiments^[5]^ showed that strain(s) of *L. cremoris* lineage 1 & 5 dominated the 4-strain blends during sequential propagation in milk, without phage predation, suggesting that these strains are the “winners” - i.e., the fittest strains for the imposed propagation regime. The fittest strains are potential targets for phage predation in accordance with the “kill-the-winner” concept^[8]^. *L. cremoris* lineage 6 and *Lc. mesenteroides* ssp. *cremoris* lineage 8 strains were the least abundant at 5% abundance for either lineage. At later sampling points, the relative abundance of genetic lineages changed further. In B-1, we observed a clear pattern of a gradual increase in abundance of the proteolytic *L. cremoris* lineage 1 & 5 strains, reaching 57.5% and 86.5% at 405 and 538 generations, respectively. Consequently, all other lineages decreased in abundance, with the biggest drop of prt^+^ *L. cremoris* lineage 3 strains by 37 percentage points between 272 and 538 generations. The prolonged propagation experiment led to two different final outcomes (i.e., alternative states) in the phage treated blends. After 538 generations, blend B-1 was dominated by *L. cremoris* lineage 1 & 5 strains and resembled the control blends in composition. In the case of replicate B-2, however, genetic *L. cremoris* lineage 1 & 5 remained at extremely low levels in the community and *L. cremoris* lineage 3, the other prt^+^ lineage in the culture, became the most abundant. These results show the enormous impact of lytic bacteriophages on population dynamics at the level of genetic lineages in defined multi-strain blends but also the conservation of one of the basic functionalities - proteolytic activity - at the community level.

### Bacteriophage resistance before, during and after propagation

**Figure 3:**
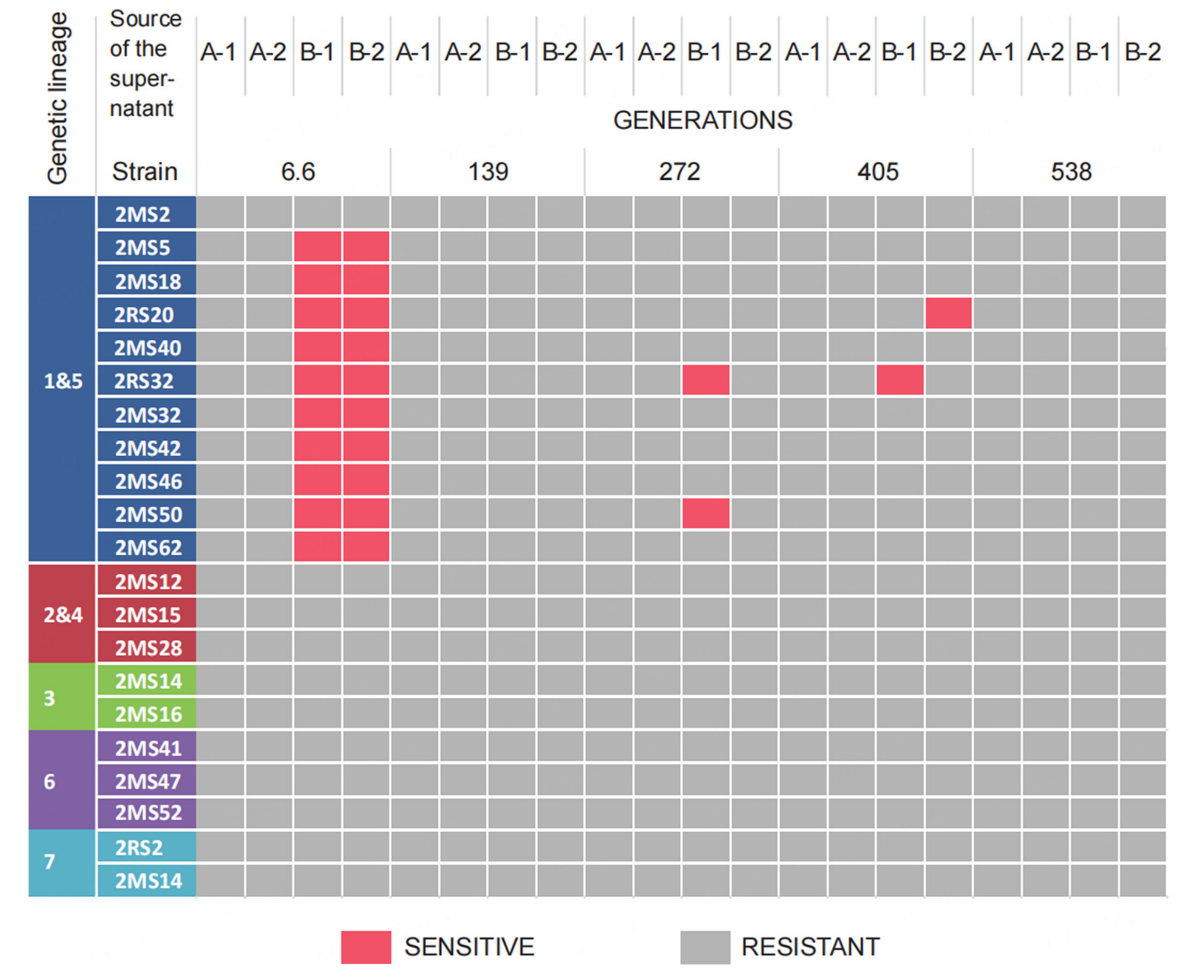
Susceptibility of the original strains used in the blend to phages present in the supernatants at different time points (after 6.6, 139, 272, 405 and 538 generations) of the propagation experiment. Blends were sequentially propagated in milk for up to 538 generations. Replicates A-1 and A-2 were used as control where no phage cocktail was added. Replicates B-1 and B-2 were challenged at the onset of the experiment with a cocktail of lactococcal phages ϕTIFN1, ϕTIFN5 and ϕTIFN7.

All 24 strains included in the blend design were individually challenged with the supernatant of the propagated blends collected at different time points of propagation to monitor the presence of lytic bacteriophages during the course of the experiment [Figure 3]. In the supernatant after 6.6 generations, we found bacteriophages predating on 10 different *L. cremoris* lineage 1 & 5 strains. Two strains, which previously showed susceptibility to phages used in the cocktail, namely 2MS2 (*L. cremoris* lineage 1 & 5) and 2MS47 (*L. cremoris* lineage 6), did not show susceptibility to phages from the supernatants of the phage-challenged blends after 6.6 generations. *L. cremoris s*train RS20, for which initially no detectable susceptibility was found to any of the three phages, showed sensitivity to phages from the supernatant of both replicates of the phage-challenged blends after 6.6 generations. Supernatants collected from the phage-challenged cultures after prolonged propagation showed lytic activity on two of the original isolates after 272 generations (2RS32 and 2MS50) and on one for each blend after 405 generations (B-1 - 2RS32 and B-2 - 2RS20) [Figure 3]. None of the supernatants collected after 139 and 538 generations caused lysis of any of the original strains.

**Figure 4:**
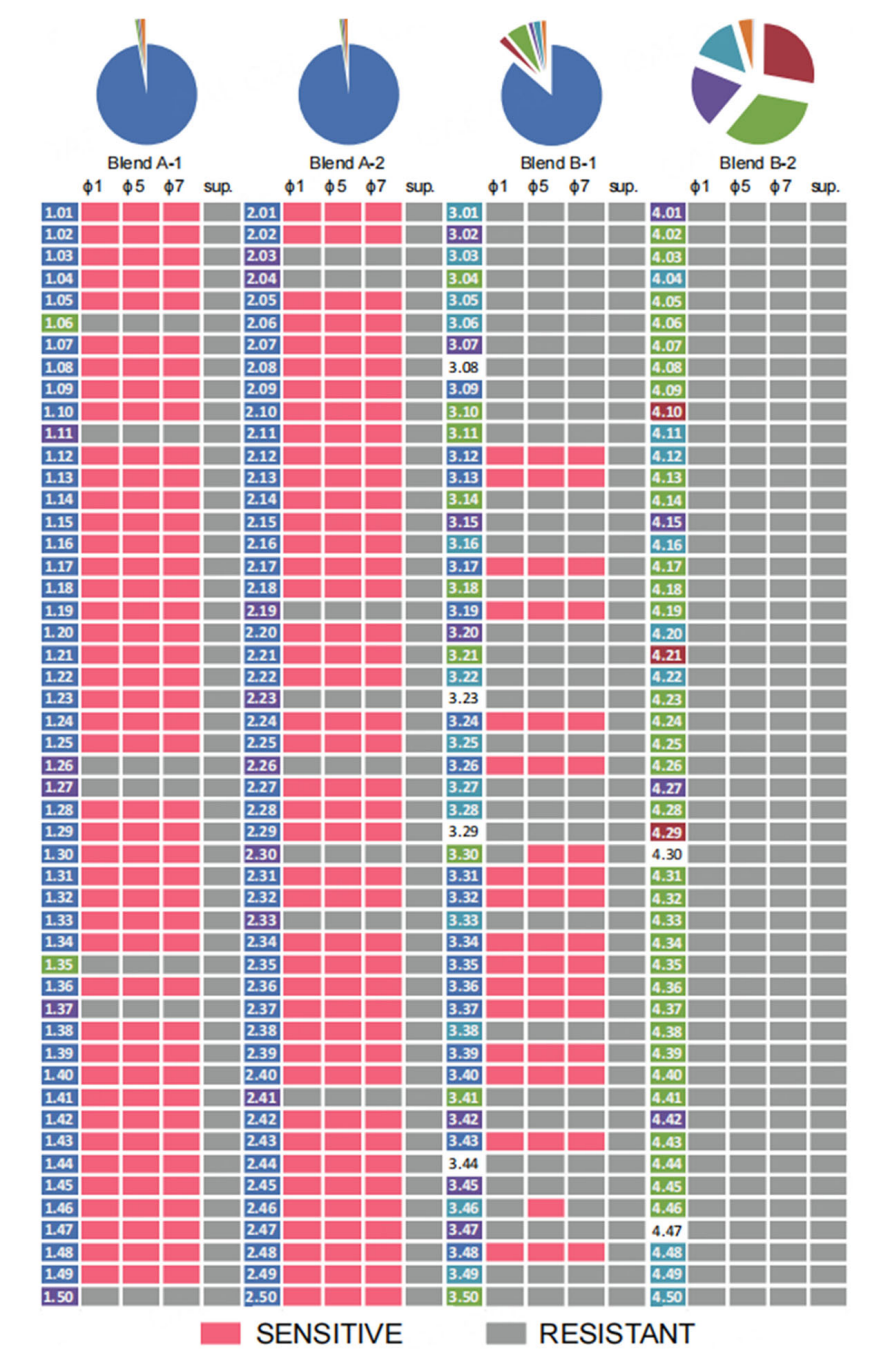
Susceptibility of the single colony isolates from the end time point of the propagation experiment to the three lactococcal phages used in the cocktail: ϕTIFN1 (ϕ1), ϕTIFN5 (ϕ5), ϕTIFN7 (ϕ7) and to the phages present in the supernatant of the blends at the end time point (538 generations). Replicates A-1 and A-2 were used as control - no phage cocktail was added. Replicates B-1 and B-2 were challenged at the onset of the experiment with a cocktail of three lactococcal phages: ϕTIFN1, ϕTIFN5 and ϕTIFN7. The strains 1.01 to 1.50 were isolated from blend A-1, strains 2.01 to 2.50 were isolated from blend A-2, strains 3.01 to 3.50 were isolated from blend B-1 and strains 4.01 to 4.50 were isolated from blend B-2 (for a detailed description see Materials and Methods section).

Next, we isolated single colonies (50 for each blend, in total 200 colony isolates) from the end time point of the propagation experiment to check if any of the previously resistant strains had become sensitive to phages used in the cocktail or to the ones possibly present in the supernatant of the cultures at the end time point. First, we determined for these 200 strains the genetic lineage identity using colony PCR^[5]^. Next, all these single colony isolates were challenged in a spot assay with individual phages used in the cocktail as well as with the supernatants of the end time point (538 generations) blends [Figure 4]. We did not find any susceptibility of strains isolated from any of the four blends A-1, A-2, B-1 and B-2 when the supernatants of the end time point were used in the spot assay. However, 18 out of 50 strains isolated from blend B-1 at the end time point were susceptible to at least one of the three phages used in the cocktail at the onset of the propagation. Most of these susceptible strains belong to *L. cremoris* lineage 1 & 5 (16/18). Two isolated strains, namely 4.30 (ascribed to *L. cremoris* lineage 3) and 4.46 (ascribed to *L. cremoris* lineage 7), were found to be susceptible to phage ϕTIFN5 and ϕTIFN7 (strain 4.30) and phage ϕTIFN5 (strain 4.46). At the onset of the experiment, the strains of *L. cremoris* lineages 3 and 7 used for the blend design were not susceptible to any of the phages used in the cocktail, showing the development of phage sensitivity in some strains during the propagation experiment or induction of a lysogenic phage, which is a known phenomenon for the *Ur* culture strains^[28]^.

Strains isolated from blend B-2 at the end time point were all shown to be resistant to the three phages. None of the strains isolated from B-2 was shown to belong to *L. cremoris* lineage 1 & 5 and most of them were ascribed to *L. cremoris* lineage 3, and did not exhibit any obvious phage sensitivity.

In terms of the strains isolated from the control blends, most of them belong to *L. cremoris* lineage 1 & 5, which in the case of blend A-1 was 43/50 strains and for blend A-2 42/50. Those 85 out of 100 strains were susceptible to all three phages used in the cocktail at the onset of propagation.

These results confirm the dynamic character of interactions of strains in blends with bacteriophages from the cocktail. In cases of control blends, strains present after 538 generations were still susceptible to the three phages used in the cocktail. Susceptible strains can thrive in the community in the absence of phage predation pressure. In contrast, in the case of the phage-challenged blend B-1, after prolonged propagation, most of the isolated strains were resistant to phages used in the cocktail at the onset of the experiment [Figure 4], demonstrating an important competitive advantage of phage resistant strains in the culture. In blend B-2, no susceptibility to phages used in the cocktail was found among strains isolated at the end time point, suggesting a lack of susceptible host and subsequent “wash out” of the lytic phages.

## DISCUSSION

This study provides data on lytic bacteriophages affecting the composition of microbial communities with genetic diversity at the level of genetic lineages. The compositional changes in the microbial community upon prolonged propagation will be discussed in the context of PS and CD community dynamics models. As indicated previously^[4]^, the relative abundance of the individual strains belonging to a specific genetic lineage of the starter culture may vary due to changes in the environment (temperature, pH) and propagation regime. In the present study, we investigated the impact of bacteriophage predation on the culture’s population dynamics at the level of co-existing genetic lineages. To reach that goal, we used our current knowledge of the structure and properties of a complex dairy starter culture *Ur* to design a defined multi-strain starter culture (blend) and sequentially propagate it in milk for an extended period of time (538 generations). The blend included all eight genetic lineages of the *Ur* starter represented by 24 strains, which extends our previous studies in which blends with three lineages represented by four strains were used^[5]^. We purposely selected strains belonging to *L. cremoris* lineage 1 & 5 - containing the “winner” (the fittest) strains - which express a diverse susceptibility to the three phages used in the cocktail. We did not observe detectable susceptibility (except strain 2MS47, *L. cremoris* lineage 6) to the three phages for the remaining strains representing *L. lactis* lineages 2 & 4, *L. cremoris* 3, 6, 7 and *Lc. mesenteroides* ssp. *cremoris* lineage 8 [Figure 1].

According to our previous work^[5]^, strains of the prt^+^ *L. cremoris* lineages 1 & 5 include the fittest strains (highest growth rate in milk supplemented with casitone) covering a diverse sensitivity profile to predation of lytic phages isolated from *Ur*. Our present results on the population dynamics in phage-challenged blends provide evidence for the prediction that the fittest strains are selected against bacteriophage predation pressure (in B-1 until 139 generations, in B-2 throughout the experiment).

Several possible events may occur during the propagation of the blend: (i) prophage induction; (ii) resistant variants could emerge and gradually increase in abundance; (iii) bacteriophages without a host would be “washed out” from the culture due to daily dilution; and (iv) evolved phages could emerge that predate on previously resistant strain(s). All of these four events would shift the rules of population dynamics towards PS or CD mechanisms and eventually impact the diversity of such a community. Based on the results obtained in the current study, we will discuss the role of phage predation as the trigger causing a catastrophic shift in the culture’s community that may finally lead to two alternative stable states.

Even though evidence exists that strains of the *Ur* starter contain inducible prophages^[28]^, we did not observe the susceptibility of *Ur* strains used to construct the blends to the supernatants of control blends (throughout the propagation experiment) and phage-treated blends (at the end time point of propagation). Based on this, we exclude the induction of an active phage crop as one of the major events impacting the population dynamics in our blends. Perhaps, even though phage particles are released by the strains, no lysis of the host occurs as described previously^[29]^.

**Figure 5:**
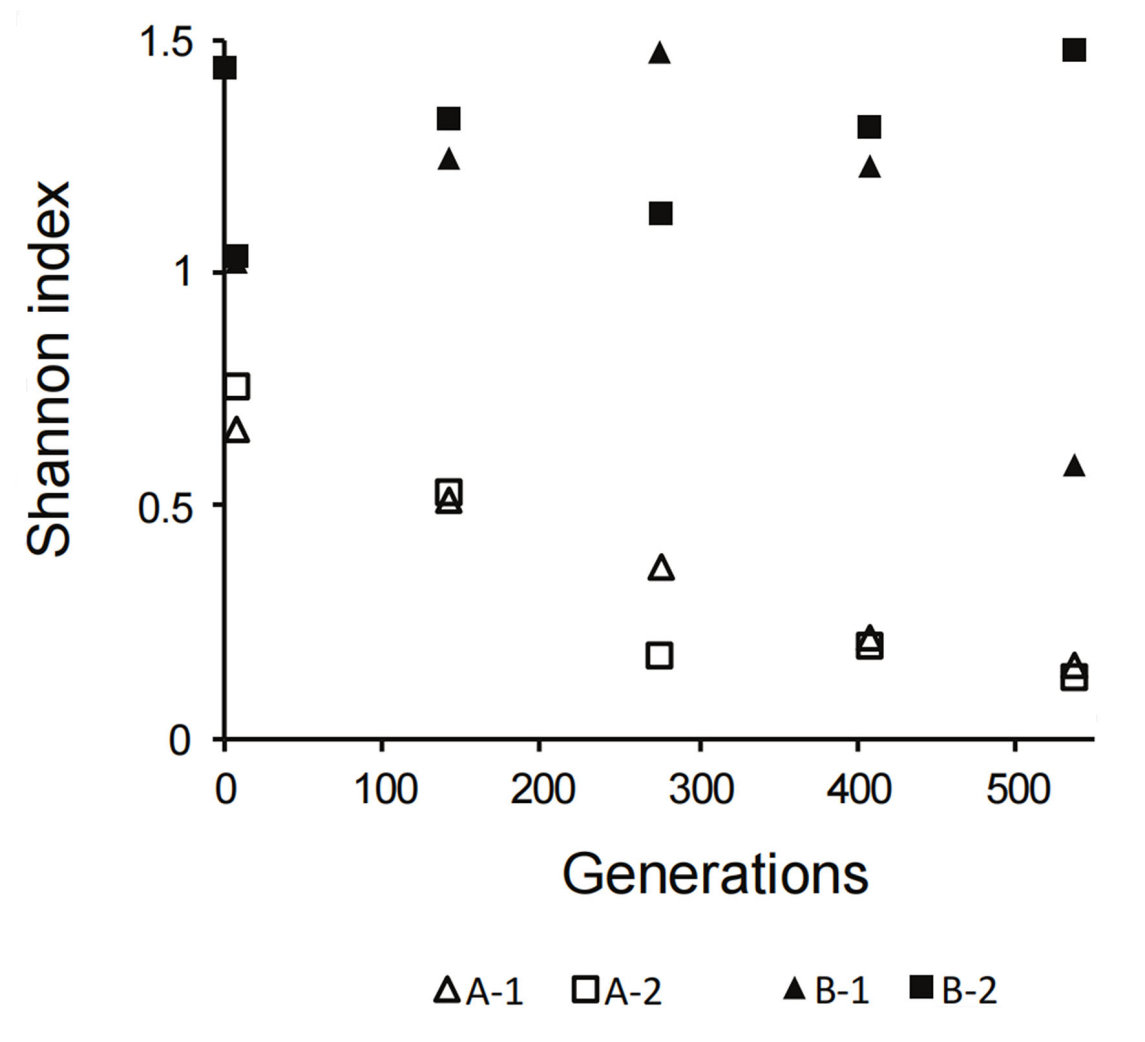
Shannon diversity index at different time points of propagation. Shannon index was calculated based on the relative abundance of genetic lineages. Open triangles represent blend A-1 (first biological replicate without phage addition); open squares represent blend A-2 (second biological replicate without phage addition); closed triangles represent blend B-1 (first biological replicate with phage addition); closed squares represent blend B-2 (second biological replicate with phage addition).

In the phage-challenged blends (B-1 and B-2), the final outcome of the propagation experiment was very different in each of the two replicates. In the case of replicate B-1, phage predation caused an initial drop in the abundance of *L. cremoris* lineage 1 & 5 strains, but recovery and a gradual increase were noted after 139 generations up to the end of the experiment. This increase can be explained by the emergence of a phage-resistant strain(s) and/or by “wash out” of phages due to the serial dilution during sequential propagation leading to an increased abundance of the faster-growing phage-resistant strain(s). We found lytic phages in the supernatant of blend B-1 after 405 generations, indicating the presence of susceptible strain(s) at that point of the experiment. Despite the presence of predators at the 405 generations time point, the abundance of *L. cremoris* lineage 1 & 5 still increased until the end time point of propagation for 538 generations, where we did not observe bacteriophage predation on the strains present in the blend anymore [Figure 4]. The absence of bacteriophage predation at the 538 generations time point, conceivably due to “wash out” between 405 and 538 generations time point, suggests that there was no phage host present anymore in the culture allowing the population of the fastest multiplying originally-present phage-resistant strains of *L. cremoris* lineage 1 & 5 [Figure 1] to increase their abundance and thus gradually become the dominant lineage in the culture, similar to the control blends. Notably, the B-1 blend (and both control blends A-1 and A-2) showed a behavior characteristic of the PS dynamics^[9]^. In conditions of absence of bacteriophage predation, the fastest multiplying strain(s) became dominant, resulting in a reduction of diversity at the level of the genetic lineage [Figure 5]. The fittest strains of *L. cremoris* lineage 1 & 5 were originally present in all blends but only in the case of lack of phage predation (blends A-1, A-2 and B-1 after 139 generations) they outcompeted less fit strains (PS dynamics).

In the case of blend B-2, strains belonging to *L. cremoris* lineage 1 & 5 did not recover from the phage attack, and after 139 generations, their relative abundance was still lower than 1%. Despite our previous observations of the relatively low growth rate of strains representing *L. lactis* lineage 2 & 4^[5]^, lineage 2 & 4 was found to be the most abundant in the B-2 blend between 139 and 405 generations. After 538 generations, the dominant prt^+^ genetic lineage in the blend (*L. cremoris* lineage 3, fourth highest growth rate for the representative strain, according to Spus *et al.*) became the most abundant^[5]^. The absence of recovery of *L. cremoris* lineages 1 & 5 is conceivably best explained by niche occupation and functional replacement by phage-resistant prt^+^ *L. cremoris* lineage 3 strains. Since *L. cremoris* lineages 1 & 5 strains were initially the dominant proteolytic lineages in the blends, the replacement by *L. cremoris* lineage 3 allows the maintenance of the proteolytic activity as a key functionality in the microbial community.

In blend B-2, culture diversity at the level of genetic lineages (expressed through the Shannon diversity index) was always higher than that of the controls [Figure 5], and we suggest that this was attributed to the initial phage predation event resulting in a decrease in the abundance of the fittest *L. cremoris* lineage 1 & 5, which remained at a low relative abundance level. Our phage resistance (spot) tests showed the absence of phages in the supernatant after 538 generations predating on the contemporary strains in the phage-challenged blends, which is in contrast to the assumption of the CD dynamics model of the constant bacteriophage predation pressure^[9]^.

**Figure 6:**
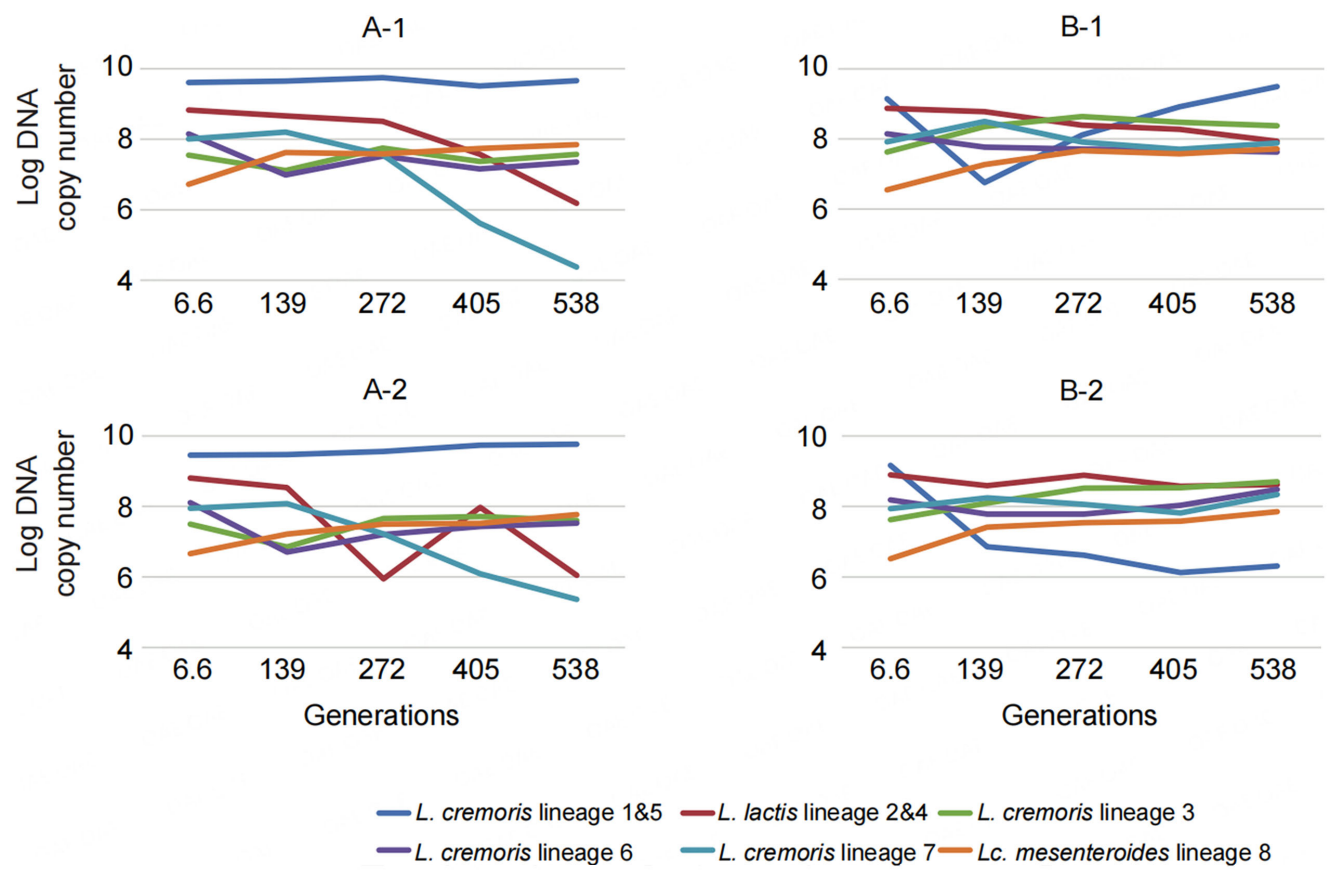
Log transformed DNA copy numbers of genetic lineages of blends A-1, A-2, B-1 and B-2 in the multi-strain blend throughout the propagation experiment. Replicates A-1 and A-2 were used as control - no phage cocktail was added. Replicates B-1 and B-2 were challenged at the onset of the experiment with the cocktail of three phages.

Although many strains were included in our model and a cocktail of three phages was used, the multi-strain blend does not represent the entire complexity of the *Ur* starter culture in which genetic lineages are assumed to be stabilized by “kill-the-winner”^[3]^. Within the time frame of the propagation experiment, the system of the multi-strain blend was not stable in terms of the relative abundance of the genetic lineages. To solve this instability, one could (i) develop a more complex model similar to the *Ur* starter; (ii) use a different initial abundance of genetic lineages; or (iii) change the propagation regime. Notably, in an evolution experiment including 186-generation-long propagation using the original *Ur* starter^[4]^, including all identified lineages and lytic phages, the clonal sweep was not observed providing additional support for the CD dynamics present in this complex cheese starter. Despite the relative instability in our model blend of strains, it is important to note that under the given conditions (defined propagation regime, presence/absence of phages, prolonged sequential propagation in milk), none of the genetic lineages was lost [Figure 6]. At the same time, due to the phage pressure, several of the sensitive strains may have been lost, and thus diversity on the strain level may have been reduced. Although interesting, we did not have the tool to investigate that. The lack of loss of the genetic lineages confirms inherent dependencies between strains in the community - microbe-microbe interactions as discussed before by Smid and Lacroix^[30]^. For example, as was suggested earlier^[5]^, the plasmid-encoded protease activity can be lost upon propagation, resulting in a fraction of prt^-^ “cheaters”^[31]^. The sub-population of “cheaters” can benefit from the peptides released due to the action of the prt^+^ fraction without carrying the burden of expressing a protease, which allows them to persist in the community. Another example of dependencies between strains is the conversion of glutamate to succinic acid, which is predicted to be a result of the combined activities of *L. lactis* and *Lc. mesenteroides*^[3]^.

It was noted in other ecosystems that the decrease in diversity reduces the optimal utilization of resources present^[32]^. The absence of bacteriophage predation pressure in the blends led to domination by strains of one particular genetic lineage (the fittest strains) without a complete clonal sweep of other lineages [Figure 6]. Possibly these low abundant strains, as specialists with, e.g., citrate degradation ability, remained in the blend by exploiting different (micro)niches. In such cases, a culture dominated by a single lineage not necessarily has lower ecosystem efficiency but, due to division of labor, exploits the resources optimally. It would be of interest to investigate the parameters of the blend efficiency (acidification, aroma formation) and to define the effect of reduction in genetic lineage diversity on the blend’s functionality.

In our propagation experiment, we observed two contrasting stable states. In the case of the absence of bacteriophage predation, the strains belonging to *L. cremoris* lineage 1 & 5 dominated the blend throughout the entire time span of the propagation. Without bacteriophage predation pressure, the strain abundance of “the winner” was not kept in control. On the other hand, the presence of phage predation led to a maintenance of diversity at the level of genetic lineages by preventing domination of the otherwise fittest strains.

In conclusion, phage predation, among other factors impacting complex microbial communities, can trigger catastrophic shifts in the community population dynamics. Nevertheless, as shown with our extremely detailed genetic lineage level culture composition analysis, recovery to an alternative stable state (demonstrating PS dynamics) can take place even after a catastrophic shift. Furthermore, it is demonstrated experimentally that bacteriophage predation on a complex microbial community can lead to prolonged stabilization of culture diversity and thus functionality as indicated by a sustained relatively high Shannon diversity index in one of the phage-challenged mixed cultures. Empirical data on the impact of bacteriophage predation on multi-strain community diversity presented in this study add a further level of detail to the PS *vs*. CD dynamics models^[9]^ describing diversity in microbial communities and stress the role of microbe-microbe interactions, bacteriophage predation and (micro)niche adaptation in preventing clonal sweeps.

## DECLARATIONS

### Availability of data and materials

Raw data were stored in the TIFN database (https://www.tifn.nl), which is accessible upon request.

### Copyright

© The Author(s) 2023.
